# Involvement of Lysosomal Labilisation and Lysosomal/mitochondrial Cross-Talk in Diclofenac Induced Hepatotoxicity

**Published:** 2011

**Authors:** Jalal Pourahmad, Yassar Mortada, Mohammad Reza Eskandari, Jafar Shahraki

**Affiliations:** a *Faculty of Pharmacy, Shahid Beheshti University of Medical Sciences, P.O. Box 14155-6153, Tehran , Iran.*; b ^b^*Faculty of Pharmacy, Shahid Beheshti University of Medical Sciences, P.O. Box 14155-6153, Tehran , Iran.*; c ^c^*Department of Pharmacology and Toxicology, School of Pharmacy, Zanjan University of Medical Sciences, Zanjan, Iran.*; d *Faculty of Pharmacy, Shahid Beheshti University of Medical Sciences, P.O. Box 14155-6153, Tehran, Iran.*

**Keywords:** Diclofenac, Cytochrome P450, Cytotoxicity, Mitochondria, Lysosome.

## Abstract

In this research, we investigated the cytotoxic mechanisms of one of the widely used pharmaceuticals that are regularly associated with the adverse effects on the liver, sometimes leading to acute liver failure, diclofenac. Diclofenac liver cytotoxicity was associated with reactive oxygen species (ROS) formation and lipid peroxidation which were inhibited by antioxidants and ROS scavengers, ferric chelator, inhibitors of reduced CYP2E1 and CYP2C9, mitochondrial permeability transition (MPT) pore sealing agents and endocytosis inhibitors. Incubation of hepatocytes with diclofenac caused rapid hepatocyte glutathione (GSH) depletion which is another marker of cellular oxidative stress. Most of the diclofenac-induced GSH depletion could be attributed to the expulsion of GSSG. Diclofenac cytotoxicity was also associated with mitochondrial injury, lysosomal membrane rupture and release of digestive proteases which were prevented by antioxidants, MPT pore sealing agents, lysosomotropic agents and inhibitors of cytochrome P450 isoenzymes. These events could cause cytochrome C release from the mitochondrial intramembrane space to cytosol. The cytochrome C release could trigger activation of caspase-3 and apoptosis. We finally concluded that diclofenac hepatotoxicity is a result of metabolic activation by CYP2E1 and CYP2C9 and ROS formation, leading to a mitochondrial/lysosomal toxic cross-talk in the liver hepatocytes.

## Introduction

Diclofenac, an arylacetic non-steroidal anti-inflammatory drug (NSAID), is frequently prescribed in treatment of rheumatic diseases as an analgesic (1, 2). NSAIDs are responsible for up to 25% of all the reported adverse drug reactions (3). Hepatotoxicity is one of the adverse reactions caused by diclofenac; it is mild to severe in a small but significant number of patients (4-6). Hepatotoxicity has been possibly resulted from the metabolism of the drug (7-9). Diclofenac-induced hepatotoxicity may involve metabolic activation to reactive the intermediates. Diclofenac was found to form the protein adducts in the liver of treated mice and rats (10, 11) as well as in hepatocyte cultures (12-14).

It has also been suggested that P450-mediated metabolism of diclofenac may be related to the apoptotic effect of the drug (1). Diclofenac has been shown to bind covalently to various liver proteins when metabolically activated by CYP2C9 (11, 15). The formation of reactive metabolite(s) by drug oxidation, which is related to drug toxicity, has already been reported (16). Diclofenac undergoes ring hydroxylation catalyzed by CYP2C9 to form its major oxidative metabolite, 4’-hydroxydiclofenac (17). Other minor product of oxidative metabolism catalyzed through CYP3A4 includes 5-hydroxydiclofenac (15, 18). Diclofenac is extensively metabolized in the liver, under two major pathways (hydroxylation and glucuronidation) in both humans and experimental animals (17). Diclofenac reactive metabolites are responsible for reduction of cytosolic O2 and generating ROS (16-17). Oxidative stress, in turn, constitutes an important risk factor for tissue damage and organ dysfunction (*e.g. *mitochondria and lysosomes).

Most of the previous investigations were performed in different cell lines and the whole mechanistic picture involved in diclofenac toxicities is yet poorly understood. Moreover, since most of the consequences of ROS formation in diclofenac cytotoxicity have not yet been completely elucidated, more detailed studies are needed to clarify the precise mechanism of diclofenac-induced hepatotoxicity. The major objective of this study was to determine diclofenac cytotoxic mechanisms in isolated rat hepatocytes.

## Experimental

*Chemicals*

Rhodamine 123, collagenase, bovine serum albumin (BSA), N-(2-hydroxyethyl) piperazine-N’-(2-ethanesulfonic acid) (HEPES), reduced and oxidized glutathione (GSH and GSSG), acridine orange, 2’,7’-dichlorofluorescin diacetate (DCFH-DA), trichloroacetic acid, trypan blue, heparin and diclofenac sodium were purchased from Sigma-Aldrich Co. (Taufkirchen, Germany). All other chemicals were of the highest commercial grade available.

*Animals*

Male Sprague-Dawley rats weighing 280 to 300 g were housed in ventilated plastic cages over PWI 8-16 hardwood bedding. There were 12 air changes per hour, 12 h light photoperiod (lights on at 08:00 h) and an environmental temperature of 21-23°C with a 50-60% relative humidity. The animals were fed with a normal standard chow diet and tap water *ad libitum*. All experiments were conducted according to the ethical standards and protocols approved by the Committee of Animal Experimentation of Shahid Beheshti University of Medical Sciences, Tehran, Iran.

*Isolation and incubation of hepatocytes*

Hepatocytes were obtained by collagenase perfusion of the liver and the viability was assessed by plasma membrane disruption determined by trypan blue (0.2 w/v) exclusion test (19). Cells were suspended at a density of 10^6^ Cells/mL in round-bottomed flasks rotating in a water bath maintained at 37°C in Krebs-Henseleit buffer (pH = 7.4), supplemented with 12.5 mM HEPES under an atmosphere of 10% O_2_, 85% N_2_ and 5% CO_2_. Each flask contained 10 mL of hepatocyte suspension. Hepatocytes were preincubated for 30 min prior to addition of chemicals. Stock solutions of all chemicals (×100 concentrated for the water solutions or ×1000 concentrated for the methanolic solutions) were prepared fresh prior to use. To avoid either non-toxic or very toxic conditions in this study, we used EC_50_ concentrations for diclofenac in the isolated hepatocytes. The EC_50_ of a chemical in hepatocyte cytotoxicity assessment technique (with the total 3 h incubation period) is defined as the concentration, which decreases the hepatocyte viability down to 50% following the 2 h of incubation (20). In order to determine this value for diclofenac, dose-response curves were plotted and then EC_50 _was determined based on a regression plot of three different concentrations (data and curves are not shown). To incubate diclofenac which is soluble in methanol, with the required concentration, we prepared methanolic stock solution (×1000 concentrated) and to achieve the required concentration in the hepatocytes, we added 10 μL samples of the stock solution to the 10 mL cell suspension. Ten μL of methanol did not affect the hepatocyte viability after 3 h incubation (data are not shown) 

*Cell viability*

The viability of isolated hepatocytes was assessed from the intactness of the plasma membrane as determined by the trypan blue (0.2% w/v) exclusion test (19). Aliquots of the hepatocyte incubate were taken at different time points during the 3-h incubation period. At least, 80-90% of the control cells were still viable after 3 h.

*Determination of reactive oxygen species*

To determine the rate of hepatocyte reactive oxygen species (ROS) generation induced by diclofenac, dichlorofluorescein diacetate (DCFH-DA, 1.6 μM) was added to the hepatocytes. It penetrates hepatocyte cells and becomes hydrolyzed to non-fluorescent dichlorofluorescein (DCFH). The latter then reacts with ROS to form the highly fluorescent dichlorofluorescein (DCF), which effluxes the cell. The fluorescence intensity of DCF was measured using a Shimadzu RF5000U fluorescence spectrophotometer. Excitation and emission wavelengths were 500 nm and 520 nm, respectively. The results were expressed as fluorescent intensity per 10^6^ cells (21).

*Lipid peroxidation assay*

Hepatocyte lipid peroxidation was determined by measuring the amount of thiobarbituric acid reactive substances (TBARS) formed during the decomposition of lipid hydroperoxides by following the absorbance at 532 nm in a Beckman DU-7 spectrophotometer (22).

*Intracellular GSH and extra cellular GSSG assessment*

GSH and GSSG were determined according to the spectrofluorometric method (23). Each sample was measured in quarts cuvettes using a fluorimeter set for 350 nm excitation and 420 nm emission wavelengths.

*Mitochondrial membrane potential assay*

Mitochondrial uptake of the cationic fluorescent dye, rhodamine123 (1.5 μM), has been used for the estimation of mitochondrial membrane potential. The amount of rhodamine123 remaining in the incubation medium was measured fluorimetrically using a Shimadzu RF5000U fluorescence spectrophotometer set at 490 nm excitation and 520 nm emission wavelengths. The capacity of mitochondria to take up the rhodamine123 was calculated as the difference (between control and treated cells) in rhodamine123 fluorescence. Our data were shown as the percentage of mitochondrial membrane potential collapse (%ΔΨm) in all treated (test) hepatocyte groups (24).

*Lysosomal membrane integrity assay*

Hepatocyte lysosomal membrane stability was determined from the redistribution of the fluorescent dye, acridine orange. Aliquots of the cell suspension (0.5 mL) that were previously stained with acridine orange (5 μM) were separated from the incubation medium by 1 min centrifugation at 1000 rpm. The cell pellet was then resuspended in 2 mL of fresh incubation medium. This washing process was carried out twice to remove the fluorescent dye from the media. Acridine orange redistribution in the cell suspension was then measured fluorimetrically using a Shimadzu RF5000U fluorescence spectrophotometer set at 495 nm excitation and 530 nm emission wavelengths. Lysosomal membrane damage was determined as the difference in redistribution of acridine orange from lysosomes into cytosol between treated cells and control cells at the time of preparation. Our data were shown as the percentage of lysosomal membrane leakiness in all treated (test) hepatocyte groups (25).

*Determination of proteolysis*

Proteolysis was monitored using a fluorescence assay for tyrosine release (adapted from (26)). An aliquot of the hepatocyte suspension was precipitated with an equal volume of 20% trichloroacetic acid and allowed to stand overnight at 4°C. The sample was vortexed and centrifuged in a benchtop clinical centrifuge (at 17,320×*g*) for 15 min. A volume of 1 mL aliquot of supernatant was removed and placed in a test tube to which 1 mL of 0.2% solution of 1-nitroso-2-naphthol and 1macid nitrite reagent (10 mg/mL NaNO2 in 20% HNO3) was added. The solution was vortexed, covered with parafilm and incubated at 37°C for 30 min.

A volume of 5 mL ethylene dichloride was added to the test tube, the mixture was vortexed vigorously and the sample was centrifuged for 10 min at high speed. The fluorescence of the aqueous phase was read in a Shimadzu RF5000U spectrophotometer (excitation at 460 nm and emission at 570 nm). The tyrosine content of the sample was determined from a standard curve constructed from known concentrations of tyrosine (0-100 μM).

*Determination of caspase-3 activity*

Caspase-3 activity was determined in cell lysate of hepatocytes from different treatments using “Sigma’s caspase-3 assay kit (CASP-3-C)” (27). In brief, this colorimetric assay is based on the hydrolysis of substrate peptide, Ac-DEVD-*p*NA, through caspase-3. The released moiety (*p*-nitroaniline) has a high absorbance at 405 nm. The concentration of the *p*-nitroaniline (μM) released from the substrate is calculated from the absorbance values at 405 nm or from a calibration curve prepared with defined *p*-nitroaniline solutions.

*Statistical analysis*

Levene’s test was used to check the homogeneity of variances. Data were analyzed using one-way analysis of variance (ANOVA) followed by Tukey’s HSD as the post-hoc test. Results were presented as mean ± SD of triplicate samples. The minimal level of significance chosen was p < 0.05.

## Results and Discussion

As shown in Table 1, diclofenac caused hepatocyte membrane lysis as determined by trypan blue uptake. The EC_50 _concentration found for diclofenac (*i.e.*, 50% membrane lysis in 2 h) was 200 μM. 

**Table 1:** Effect of lipid antioxidant, ROS scavengers, ferric chelator, MPT pore sealing agents, lysosomotropic agents, and CYP2E1 and CYP2C9 inhibitors on diclofenac-induced hepatocyte lysis, ROS formation and lipid peroxidation

**Addition**	**Cytotoxicity (%) 3h**	**DCF 1h**	**TBARS (nM) 3h**
None	25 ± 2	228 ± 12	460 ± 23
Diclofenac (200 μM)	70 ± 4^a^	362±18^a^	1050 ± 52^a^
+*α*-Tocopherol succinate (10 μM)	50 ± 3^b^	205 ± 10^b^	500 ± 25^b^
+Mannitol (50 mM)	40 ± 3^b^	207 ± 23^b^	320 ± 16^b^
+Dimethyl sulfoxide (150 μM)	52 ± 3^b^	219 ± 45^b^	350 ± 17^b^
+Deferoxamine (200 μM)	52 ± 5^b^	202 ± 10^b^	300 ± 15^b^
+Carnitine (2 mM)	54 ± 2^b^	199 ± 25^b^	600 ± 30^b^
+Trifluoperazine (15 μM)	54 ± 3^b^	228 ± 41^b^	510 ± 25^b^
+Cyclosporine (2 μM)	45 ± 2^b^	210 ± 22^b^	650 ± 32^b^
+Methylamine (30 mM)	55 ± 4^b^	210 ± 27^b^	570 ± 28^b^
+Chloroquine (100 μM)	51 ± 5^b^	225 ± 26^b^	400 ± 20^b^
+Phenylimidazole (300 μM)	52 ± 4^b^	213 ± 21^b^	570 ± 28^b^
+Diphenyliodonium chloride(50 μM)	50 ± 5^b^	220 ± 20^b^	600 ± 30^b^
+4-methylpyrazole (500 μM)	51 ± 5^b^	235 ± 26^b^	650 ± 33^b^
+sulfaphenazole (60 μM)	51 ± 5^b^	228 ± 26^b^	630 ± 35^b^
+GSH (2 mM)	39 ± 3^b^	242 ± 38^b^	523 ± 25^b^

Hepatocytes (10^6 ^Cells/mL) were incubated in Krebs–Henseleit buffer with pH of 7.4 at 37°C for 3.0 h following the addition of diclofenac. Cytotoxicity was determined as the percentage of cells that uptake trypan blue (Chan *et al.*, 2008). DCF formation was expressed as fluorescent intensity units (Pourahmad *et al*., 2010). TBARS formation was expressed as nM concentrations (Jamshidzadeh *et al.*, 2007). Values are expressed as mean ± SD of three separate experiments (n = 3). ^a^: Significant difference in comparison with control hepatocytes (p < 0.05); ^b^: Significant difference in comparison with diclofenac treated hepatocytes (p < 0.05)

In addition, when hepatocytes were incubated with diclofenac at this EC_50_ concentration, ROS formation determined by the oxidation of dichlorofluorescein diacetate to dichlorofluorescein was significantly (p < 0.05) increased. In addition, a significant amount (p < 0.05) of thiobarbituric acid reactive substances (TBARS) was formed. As shown in Table 1, the TBARS concentrations when hepatocytes were incubated with diclofenac markedly increased in the 3^rd^ h. Diclofenac-induced cytotoxicity, TBARS and ROS generation were prevented by lipid antioxidant (*α*-Tocopherol succinate), hydroxyl radical scavengers (mannitol, dimethyl sulfoxide), ferric chelator (deferoxamine), MPT pore sealing agents (carnitine, trifluoperazine, cyclosporine), lysosomotropic agents (methylamine, chloroquine), NADPH P450 reductase inhibitor (diphenyliodonium chloride), CYP2E1 inhibitors (phenylimidazole, 4-methylpyrazole) and CYP2C9 inhibitor (sulfaphenazole). Diclofenac-induced cytotoxicity, ROS formation and lipid peroxidation were also inhibited by GSH (Table 1). All of these protective agents did not show any toxic effects on hepatocytes at concentrations used (data not shown).

As shown in Table 2, diclofenac induced a rapid decline of hepatocyte mitochondrial membrane potential which was prevented by lipid antioxidant (*α*-Tocopherol succinate), hydroxyl radical scavengers (mannitol, dimethyl sulfoxide) and ferric chelator (deferoxamine) indicating that the decline of mitochondrial membrane potential was a consequence of ROS formation and lipid peroxidation. In addition, lysosomotropic agents (methylamine, chloroquine), NADPH P450 reductase inhibitor (diphenyliodonium chloride), CYP2E1 inhibitors (phenylimidazole, 4-methylpyrazole) and CYP2C9 inhibitor (sulfaphenazole) repressed decline of mitochondrial membrane potential (Table 2). All of these reagents did not show any significant changes on hepatocytes mitochondrial membrane potential at concentrations used (data not shown).

**Table 2:** Mitochondrial membrane potential change during the diclofenac-induced hepatocyte injury

**Addition**	**%ΔΨm**			
	**Incubation time**			
	**15 min**	**30 min**	**60 min**	**120 min**
Diclofenac (200 μM)	10 ± 1	12 ± 1	22 ± 2	30 ± 2
+*α*-Tocopherol succinate (10 μM)	6 ± 1^a^	5 ± 1^a^	10 ± 2^a^	12 ± 1^a^
+Mannitol (50 mM)	5 ± 1^a^	7 ± 1^a^	10 ± 2^a^	15 ± 1^a^
+Dimethyl sulfoxide (150 μM)	3 ± 1^a^	8 ± 1^a^	9 ± 1^a^	12 ± 1^a^
+Deferoxamine (200 μM)	5 ± 1^a^	8 ± 1^a^	12 ± 2^a^	18 ± 1^a^
+Carnitine (2 mM)	2 ± 1^a^	8 ± 1^a^	10 ± 2^a^	15 ± 1^a^
+Trifluoperazine (15 μM)	4 ± 2^a^	5 ± 2^a^	6 ± 2^a^	10 ± 1^a^
+Cyclosporine (2 μM)	3 ± 1^a^	7 ± 1^a^	9 ± 2^a^	17 ± 1^a^
+Methylamine (30 mM)	7 ± 1^a^	8 ± 1^a^	11 ± 2^a^	16 ± 1^a^
+Chloroquine (100 μM)	6 ± 1^a^	7 ± 1^a^	9 ± 2^a^	14 ± 1^a^
+Phenylimidazole (300 μM)	7 ± 1^a^	8 ± 1^a^	10 ± 1^a^	13 ± 1^a^
+Diphenyliodonium chloride(50 μM)	6 ± 1^a^	7 ± 1^a^	9 ± 1^a^	11 ± 1^a^
+4-methylpyrazole (5 00μM)	5 ± 1^a^	6 ± 2^a^	8 ± 1^a^	10 ± 1^a^
+sulfaphenazole (60 μM)	6 ± 1^a^	7 ± 2^a^	9 ± 1^a^	11 ± 1^a^

Hepatocytes (10^6^ Cells/mL) were incubated in Krebs–Henseleit buffer pH of 7.4 at 37°C. Mitochondrial membrane potential was determined as the difference in mitochondrial uptake of the rhodamine 123 between the control and treated cells. Our data were shown as the percentage of mitochondrial membrane potential collapse (%ΔΨm) in all the treated (test) hepatocyte groups (Andersson *et al.*, 1987). Values are expressed as mean ± SD of three separate experiments (n = 3). ^a^: Significant difference in comparison with diclofenac-treated hepatocytes (p < 0.05)

When hepatocyte lysosomes were loaded with acridine orange (a lysosomotropic agent), a significant release of acridine orange was ensued into the cytosolic fraction within 120 min of incubation with diclofenac indicating a severe damage to lysosomal membrane (Table 3). 

**Table 3:** Lysosomal membrane integrity changes during the diclofenac-induced hepatocyte injury

**Addition**	**% Acridine orange redistribution**			
	**Incubation time**			
	**15 min**	**30 min**	**60 min**	**120 min**
Diclofenac (200 μM)	14 ± 1	20 ± 1	25 ± 1	30 ± 2
+α-Tocopherol succinate (10 μM)	1 ± 0.1^a^	16 ± 1^a^	17 ± 1^a^	21 ± 1^a^
+Mannitol (50 mM)	5 ± 0.5^a^	8 ± 1^a^	12 ± 1^a^	15 ± 2^a^
+Dimethyl sulfoxide (150 μM)	5 ± 0.5^a^	7 ± 1^a^	10 ± 1^a^	13 ± 1^a^
+Deferoxamine (200 μM)	4 ± 0.2^a^	8 ± 1^a^	13 ± 1^a^	16 ± 2^a^
+Carnitine (2 mM)	10 ± 1^a^	11 ± 1^a^	13 ± 2^a^	15 ± 1^a^
+Trifluoperazine (15 μM)	2 ± 0.2^a^	10 ± 2^a^	11 ± 3^a^	14 ± 3^a^
+Cyclosporine (2 μM)	9 ± 1^a^	10 ± 1^a^	12 ± 2^a^	13 ± 1^a^
+Methylamine (30 mM)	2 ± 0.2^a^	7 ± 1^a^	9 ± 1^a^	13 ± 1^a^
+Chloroquine (100 μM)	1 ± 0.1^a^	10 ± 1^a^	14 ± 1^a^	15 ± 1^a^
+Phenylimidazole (300 μM)	5 ± 0.5^a^	13 ± 1^a^	16 ± 1^a^	20 ± 1^a^
+Diphenyliodonium chloride(50 μM)	6 ± 1^a^	7 ± 1^a^	9 ± 1^a^	11 ± 1^a^
+4-methylpyrazole (500μM)	5 ± 1^a^	6 ± 2^a^	8 ± 1^a^	9 ± 1^a^
+sulfaphenazole (60 μM)	7 ± 1^a^	9 ± 2^a^	11 ± 2^a^	12 ± 1^a^
+GSH (2 mM)	4 ± 0.5^a^	9 ± 2^a^	11 ± 1^a^	11 ± 3^a^

Hepatocytes (10^6^ Cells/mL) were incubated in Krebs–Henseleit buffer with pH of 7.4 at 37°C. Lysosomal membrane damage was determined as the difference in redistribution of acridine orange from lysosomes into cytosol between the treated cells and control cells. Our data were shown as the percentage of lysosomal membrane leakiness in all the treated (test) hepatocyte groups (Pourahmad *et al.*, 2009). Values are expressed as mean ± SD of three separate experiments (n = 3). ^a^: Significant difference in comparison with diclofenac-treated hepatocytes (p < 0.05)

Diclofenac-induced acridine orange release was again prevented by lipid antioxidant (*α*-Tocopherol succinate), hydroxyl radical scavengers (mannitol, dimethyl sulfoxide), ferric chelator (deferoxamine), MPT pore sealing agents (carnitine, trifluoperazine, cyclosporine), NADPH P450 reductase inhibitor (diphenyliodonium chloride), CYP2E1 inhibitors (phenylimidazole, 4-methylpyrazole), CYP2C9 inhibitor (sulfaphenazole) and GSH (Table 3). All of these reagents did not show any effects on acridine orange redistribution from lysosomes to cytosol at concentrations used (data not shown). 

Hepatocyte proteolysis as determined by the release of the amino acid tyrosine into the extracellular medium over 120 min was markedly increased when hepatocytes were incubated with diclofenac (Table 4). Diclofenac-induced tyrosine release was prevented by the lipid antioxidant (*α*-Tocopherol succinate), hydroxyl radical scavengers (mannitol, dimethyl sulfoxide), ferric chelator (deferoxamine), MPT pore sealing agents (carnitine, trifluoperazine, cyclosporine), lysosomotropic agents (methylamine, chloroquine), NADPH P450 reductase inhibitor (diphenyliodonium chloride), CYP2E1 inhibitors (phenylimidazole, 4-methylpyrazole), CYP2C9 inhibitor (sulfaphenazole) and GSH (Table 4). All of these reagents did not show any effects on tyrosine release at concentrations used (data not shown).

**Table 4:** Effect of lipid antioxidant, ROS scavengers, ferric chelator, MPT pore sealing agents, lysosomotropic agents, and CYP2E1 and CYP2C9 inhibitors on diclofenac-induced hepatocyte proteolysis.

**Addition**	**Hepatocyte tyrosine release (μM) 2h**
None	0
Diclofenac (200 μM)	12 ± 0.5^a^
+*α*-Tocopherol succinate (10 μM)	5.9 ± 0.3^b^
+Mannitol (50 mM)	5.8 ± 0.3^b^
+Dimethyl sulfoxide (150 μM)	6.2 ± 1.3^b^
+Deferoxamine (200 μM)	6.3 ± 0.5^b^
+Carnitine (2 mM)	6.6 ± 0.4^b^
+Trifluoperazine (15 μM)	8.0 ± 1.1^b^
+Cyclosporine (2 μM)	5.4 ± 0.6^b^
+Methylamine (30 mM)	7.5 ± 0.4^b^
+Chloroquine (100 μM)	6.3 ± 0.4^b^
+Phenylimidazole (300 μM)	6.6 ± 0.5^b^
+Diphenyliodonium chloride(50 μM)	6.8 ± 0.2^b^
+4-methylpyrazole (500 μM)	5.4 ± 0.5^b^
+sulfaphenazole (60 μM)	7.2 ± 0.5^b^
+GSH (2 mM)	5.1 ± 1.0^b^

Hepatocytes (10^6^ Cells/mL) were incubated in Krebs–Henseleit buffer with pH of 7.4 at 37°C for 2.0 h following the addition of diclofenac. Lysosomal damage-induced proteolysis was determined by measuring the cellular release of tyrosine into the media (Novak *et al.*, 1988). Values are expressed as mean ± SD of three separate experiments (n = 3). ^a^: Significant difference in comparison with control hepatocytes (p < 0.05); ^b^: Significant difference in comparison with diclofenac treated hepatocytes (p < 0.05)

As shown in Table 5, diclofenac significantly increased the activity of apoptosis final mediator, caspae-3. Increased caspase-3 activity was prevented by lipid antioxidant (*α*-Tocopherol succinate), hydroxyl radical scavengers (mannitol, dimethyl sulfoxide), ferric chelator (deferoxamine), MPT pore sealing agents (carnitine, trifluoperazine, cyclosporine), lysosomotropic agents (methylamine, chloroquine), NADPH P450 reductase inhibitor (diphenyliodonium chloride), CYP2E1 inhibitors (phenylimidazole, 4-methylpyrazole) and CYP2C9 inhibitor (sulfaphenazole) (Table 5). All of these mentioned agents did not significantly change caspase-3 activity at concentrations used (data not shown).

**Table 5:** Caspase-3 activity changes during the diclofenac-induced hepatocyte injury

**Addition **	**Caspase-3 activity (μM ** ***p*** **NA/mL/min**)
None	76 ± 4
Diclofenac (200 μM)	126 ± 6^a^
+*α*-Tocopherol succinate (10 μM)	65 ± 3^b^
+Mannitol (50 mM)	61 ± 3^b^
+Dimethyl sulfoxide (150 μM)	70 ± 3^b^
+Deferoxamine (200 μM)	86 ± 4^b^
+Carnitine (2 mM)	11 ± 1^b^
+Trifluoperazine (15 μM)	61 ± 3^b^
+Cyclosporine (2 μM)	10 ± 1^b^
+Methylamine (30 mM)	85 ± 4^b^
+Chloroquine (100 μM)	75 ± 3^b^
+Phenylimidazole (300 μM)	87 ± 25^b^
+Diphenyliodonium chloride(50 μM)	70 ± 15^b^
+4-methylpyrazole (500 μM)	75 ± 13^b^
+sulfaphenazole (60 μM)	68 ± 18^b^

Hepatocytes (10^6^ Cells/mL) were incubated in Krebs–Henseleit buffer with pH of 7.4 at 37°C for 2 h following the addition of diclofenac. Caspase-3 activity was determined by Sigma-Aldrich kit (Sakahira *et al.*, 1998). The kit determines produced pNA that is released from the interaction of caspase-3 and AC-DEVD-pNA (peptide substrate). Values are expressed as mean ± SD of three separate experiments (n = 3). ^a^: Significant difference in comparison with control hepatocytes (p < 0.05); ^b^: Significant difference in comparison with diclofenac treated hepatocytes (p < 0.05).

As shown in Table 6, the incubation of hepatocytes with diclofenac caused rapid hepatocyte GSH depletion. Most of the diclofenac-induced GSH depletion could be attributed to the expulsion of GSSG (Table 6). Again, lipid antioxidant (*α*-Tocopherol succinate), hydroxyl radical scavengers (mannitol, dimethyl sulfoxide), ferric chelator (deferoxamine), MPT pore sealing agents (carnitine, trifluoperazine, cyclosporine), lysosomotropic agents (methylamine, chloroquine) NADPH P450 reductase inhibitor (diphenyliodonium chloride), CYP2E1 inhibitors (phenylimidazole, 4-methylpyrazole) and CYP2C9 inhibitor (sulfaphenazole) significantly (p < 0.05) prevented both diclofenac-induced intracellular GSH decrease and extracellular GSSG increase (Table 6). All of these reagents did not show any significant effect on hepatocytes GSH/GSSG status at concentrations used (data not shown).

**Table 6:** Effect of lipid antioxidant, “ROS” scavengers, ferric chelator, lysosomotropic agents, MPT pore sealing agents, and CYP2E1 and CYP2C9 inhibitors on the diclofenac-induced glutathione depletion

**Extra cellular GSSG (μM) 3h**	**Intracellular GSH (μM) 3h**	**Addition**
5.1 ± 0.5	49 ± 4	None
11 ± 1^a^	13 ± 1^a^	Diclofenac (200 μM)
4.1 ± 0.4^b^	37 ± 3^b^	+*α*-Tocopherol succinate (10 μM)
5.5 ± 0.5^b^	28 ± 5^b^	+Mannitol (50 mM)
4.8 ± 0.4^b^	35 ± 3^b^	+Dimethyl sulfoxide (150 μM)
4.5 ± 0.4^b^	24 ± 3^b^	+Deferoxamine (200 μM)
5.1 ± 0.5^b^	25 ± 2^b^	+Carnitine (2 mM)
6.2 ± 0.6^b^	27 ± 3^b^	+Trifluoperazine (15 μM)
4.2 ± 0.5^b^	10 ± 2^b^	+Cyclosporine (2 μM)
3.6 ± 0.3^b^	26 ± 3^b^	+Methylamine (30 mM)
6.1 ± 0.6^b^	25 ± 2^b^	+Chloroquine (100 μM)
4.2 ± 0.4^b^	33 ± 5^b^	+Phenylimidazole (300 μM)
3.5 ± 0.3^b^	30 ± 5^b^	+Diphenyliodonium chloride(50 μM)
4.3 ± 0.4^b^	35 ± 5^b^	+4-methylpyrazole (500 μM)
4.1 ± 0.3^b^	31 ± 5^b^	+sulfaphenazole (60 μM)

Hepatocytes (10^6^ Cells/mL) were incubated in Krebs–Henseleit buffer with pH of 7.4 at 37°C for 3.0 h following the addition of diclofenac. Intracellular GSH and extra cellular GSSG were fluorimetrically determined as described by Hissin and Hilf, 1978. Values are expressed as mean ± SD of three separate experiments (n = 3). ^a^: Significant difference in comparison with control hepatocytes (p < 0.05); ^b^: Significant difference in comparison with the diclofenac-treated hepatocytes (p < 0.05)

Diclofenac is a widely used non-steroidal anti-inflammatory drug that has been associated with rare but serious hepatotoxic adverse reaction (28). It has been shown that oxidative stress is one of the strong candidates in diclofenac-induced hepatotoxicity and that diclofenac increases the ROS production and produces the oxidative stress in different cell lines. The mechanism suggested for NSAID drugs-induced liver toxicity was based on peroxidase catalyzed production of NSAID radicals, which in turn can oxidize GSH and NADPH and reduce cytosolic oxygen to form O_2_^.^^─^ and H_2_O_2_ radicals (29).

Metabolites of diclofenac may produce oxidative stress by either generating ROS via the redox cycling or depletion of glutathione (GSH) (29, 30). This all together may lead to liver cell necrosis and ultimately acute liver failure (12, 15, 31, and 32). Our results showed that diclofenac induces hepatocyte membrane lysis, ROS generation and lipid peroxidation. Glutathione (GSH) is an intracellular antioxidant that prevents intracellular ROS formation and lipid peroxidation. As an antioxidant, it has been involved in cell protection from the deleterious effect of oxidative stress, both directly and as a cofactor of glutathione peroxidases and these reactions generate oxidized glutathione (GSSG) (33). So, glutathione depletion is a marker of cellular oxidative stress and could be attributed to the expulsion of GSSG. Our results showed that when isolated hepatocytes were incubated with diclofenac, glutathione depletion occurred as a consequence of ROS formation and lipid peroxidation. Glutathione depletion and lysosomal membrane leakage observed in our study could also accelerate and exacerbate the oxidative stress cytotoxicity. GSH depletion can also disrupt the mitochondrial transmembrane potential and consequently cause MPT pore opening and cytochrome C release which is the initiator of apoptosis signaling in the cytosol (34).

Previous studies showed that the toxic effect of diclofenac on hepatocytes may be caused by drug-induced mitochondrial toxicity (9 and 35-37). It was also shown that diclofenac could cause mitochondrial oxidative stress (38) and MPT pore opening as a direct consequence of the diclofenac protonophoretic and uncoupling activity (39). In our study, the hepatocyte mitochondrial membrane potential was rapidly decreased by diclofenac which was prevented by lipid antioxidant, hydroxyl radical scavengers and ferric chelator indicating that mitochondrial membrane damage was a consequence of ROS formation and lipid peroxidation. The ΔΨm is maintained by continuous pumping of protons from the matrix across the inner mitochondrial membrane into the intermembrane space. Since these protons in turn are used to drive the ATP synthase, a collapse of the ΔΨm invariably results in compromised ATP synthesis. Any damage to mitochondrial ATP generation results in intracellular acidosis and osmotic injury. The latter is the cause of plasma membrane lysis (40).

ROS-induced mitochondrial damage could spread, and then hydrogen peroxide (H_2_O_2_) originated in mitochondria diffuses into lysosomes and a Fenton-type reaction (Haber-Weiss reaction) catalyzed by intralysosomal redox-active iron occurred. This reaction generates highly reactive hydroxyl radical (HO^.^) that can destabilize the lysosomal membrane integrity. As a result, digestive proteases and free radicals could be released into the cytosol. Our results showed that diclofenac-induced hepatocyte injury involved lysosomal membrane damage and release of proteolytic enzymes including cathepsins (B, D, L). These proteolytic enzymes potentiate the opening of MPT pore and cytochrome C release and also initiate the downstream events that trigger caspase-3 activation and apoptosis. ROS can also be produced intracellularly by other pathways. One of these pathways involves cytochrome P450s.

CYP2E1 is one of the most powerful inducers of oxidative stress in liver cells (41). CYP2E1 itself is also an effective enzyme for ROS production, exhibiting enhanced NADPH oxidase activity, and elevated rates of the production of O_2_^.^^─ ^and H_2_O_2_ even in the absence of substrate (42-44). Diclofenac undergoes ring hydroxylation catalyzed by CYP2C9, resulting in the formation of the major oxidative metabolite, 4’-hydroxydiclofenac (17). Our results showed that NADPH P450 reductase inhibitor (diphenyliodonium chloride), CYP2E1 inhibitors (Phenylimidazole, 4-methylpyrazole) and CYP2C9 inhibitor (sulfaphenazole) prevented diclofenac-induced cytotoxicity, ROS and TBARS generation, mitochondrial membrane damage, lysosomal membrane damage, proteolysis, caspase-3 activity and GSH depletion. It can therefore be suggested that CYP2E1 and CYP2C9 together mediated the bioactivation of diclofenac which is linked to the increased production of ROS and progression of oxidative stress.

Our other interesting results were that the lysosomotropic agents (*e.g. *chloroquine, methylamine) prevented from diclofenac-induced mitochondrial membrane potential collapse and mitochondrial MPT pore sealing agents (*e.g. *cyclosporine, carnitine, trifluoperazine) inhibited lysosomal membrane damage caused by diclofenac. It can therefore be suggested that there is probably a toxic interaction between mitochondrial and lysosomal oxidative stress generating systems, which potentiates each organelle damage and ROS formation in diclofenac liver toxicity. Metabolic activation of diclofenac through cytochrome P450 monooxygenases (*e.g. *CYP2E1 and CYP2C9) leads to NSAID radical formation (*i.e. *4’-hydroxydiclofenac and 5-hydroxydiclofenac) which can reduce the cytosolic oxygen and increase the hepatocyte ROS generation. Increased ROS formation could directly damage the hepatocyte mitochondria via MPT pore opening and disruption of electron transfer chain. Hydrogen peroxide (H_2_O_2_) originated either from diclofenac metabolic activation or damaged mitochondria diffuses into lysosomes which leads to a Fenton-type reaction (Haber-weiss) catalyzed by intralysosomal redox-active Fe^2+^/Fe^3+^. This results in a highly reactive hydroxyl radical (HO°) generation. Hydroxyl radicals could destabilize the lysosomal membrane integrity and the release of digestive proteases (*i.e. *cathepsins). These released proteases and hydroxyl radicals could either open the mitochondrial MPT pore via the oxidation of surrounding thiol groups or through the activation of Bid or Bax pro-apoptotic proteins and other lytic enzymes including phospholipase A_2 _(PLA_2_). Disruption of electron transfer chain following the efflux of cytochrome C further potentiates mitochondrial H_2_O_2_ generation and continues the cycle of mitochondrial/lysosomal toxic interaction of oxidative stress.

To sum up, Diclofenac hepatotoxicity is a result of metabolic activation by CYP2E1 and CYP2C9 and ROS formation, leading to a mitochondrial/lysosomal oxidative stress injury and glutathione depletion. Mitochondrial and lysosomal toxic interactions are probably responsible for potentiating the liver toxicity and oxidative stress. 

## References

[1] Gómez-Lechón MJ, Ponsoda X, O›Connor E, Donato T, Jover R, Castell JV (2003). Diclofenac induces apoptosis in hepatocytes. Toxicol. In-vitro.

[2] Iveson TJ, Ryley NG, Kelly PM, Trowell JM, McGee JO, Chapman RW (1990). Diclofenac associated hepatitis. J. Hepatol.

[3] Galati G, Tafazoli S, Sabzevari O, Chan TS, O›Brien PJ (2002). Idiosyncratic NSAID drug induced oxidative stress. Chem. Biol. Interact.

[4] Pourahmad J (2008). The Need to Establish a Common Base Line for the Characteristics of Safety Withdrawal of Prescription Drugs from Worldwide Pharmaceutical Markets. Iranian J. Pharm. Res.

[5] Purcell P, Henry D, Melville G (1991). Diclofenac hepatitis. Gut.

[6] Suzuki H, Inoue T, Matsushita T, Kobayashi K, Horii I, Hirabayashi Y, Inoue T (2008). In-vitro gene expression analysis of hepatotoxic drugs in rat primary hepatocytes. J. Appl. Toxicol.

[7] Schmitz G, Stauffert I, Sippel H, Lepper H, Estler CJ (1992). Toxicity of diclofenac to isolated hepatocytes. J. Hepatol.

[8] Jurima-Romet M, Crawford K, Huang HS (1994). Comparative cytotoxicity of non-steroidal anti-inflammatory drugs in primary cultures of rat hepatocytes. Toxicol. In-vitro.

[9] Bort R, Ponsoda X, Jover R, Gómez-Lechón MJ, Castell JV (1998). Diclofenac Toxicity to Hepatocytes: A Role for Drug Metabolism in Cell Toxicity. J. Pharmacol. Exp. Ther.

[10] Pumford NR, Myers TG, Davila JC, Highet RJ, Pohl LR (1993). Immunochemical detection of liver protein adducts of the non-steroidal antiinflammatory drug diclofenac. Chem. Res. Toxicol.

[11] Hargus SJ, Martin BM, George JW, Pohl LR (1995). Covalent modification of rat liver dipeptidyl peptidase IV (CD26) by the non-steroidal anti-inflammatory drug diclofenac. Chem. Res. Toxicol.

[12] Kretz-Rommel A, Boelsterli UA (1993). Diclofenac covalent protein binding is dependent on acyl glucuronide formation and is inversely related to P450-mediated acute cell injury in cultured rat hepatocytes. Toxicol. Appl. Pharmacol.

[13] Boelsterli UA, Zimmerman HJ, Kretz-Rommel A (1995). Idiosyncratic liver toxicity of non-steroidal antiinflammatory drugs: Molecular mechanisms and pathology. Crit. Rev. Toxicol.

[14] Gil ML, Ramirez MC, Terencio MC, Castell JV (1995). Immunochemical detection of protein adducts in cultured human hepatocytes exposed to diclofenac. Biochem. Biophys. Acta.

[15] Tang W, Stearns RA, Bandiera SM, Zhang Y, Raab C, Braun MP, Dean DC, Pang J, Leung KH, Doss GA, Strauss JR, Kwei GY, Rushmore TH, Chiu SH, Baillie TA (1999). Studies on cytochrome P450-mediated bioactivation of diclofenac in rats and in human hepatocytes: identification of glutathione conjugated metabolites. Drug. Metab. Dispos.

[16] Miyamoto G, Zahid N, Uetrecht JP (1997). Oxidation of diclofenac to reactive intermediates by neutrophils, myeloperoxidase and hypochlorous acid. Chem. Res. Toxicol.

[17] Stierlin H, Faigle JW (1979). Biotransformation of diclofenac sodium (Voltaren) in animals and man. II. Quantitative determination of the unchanged drug and principal phenolic metabolites, in urine and bile. Xenobiotica.

[18] Shen S, Marchick MR, Davis MR, Doss GA, Pohl LR (1999). Metabolic activation of diclofenac by human cytochrome P450 3A4: role of 5-hydroxydiclofenac. Chem. Res. Toxicol.

[19] Chan K, Jensen N, O’Brien PJ (2008). Structure–activity relationships for thiol reactivity and rat or human hepatocyte toxicity induced by substituted p-benzoquinone compounds. J. Appl Toxicol.

[20] Galati G, Teng S, Moridani MY, Chan TS, O›Brien PJ (2000). Cancer chemoprevention and apoptosis mechanisms induced by dietary polyphenolics. Drug. Metabol. Drug. Interact.

[21] Pourahmad J (2002). Identification of Intracellular Sources Responsible for Endogenous Reactive Oxygen Species Formation. IJPR.

[22] Jamshidzadeh A, Niknahad H, Kashafi H (2007). Cytotoxicity of chloroquine in isolated rat hepatocytes. J. Appl. Toxicol.

[23] Hissin J, Hilf R (1976). A fluorometric method for determination of oxidised and reduced glutathion in tissues. Anal. Biochem.

[24] Andersson BS, Aw TY, Jones DP (1987). Mitochondrial transmembrane potential and pH gradient during anoxia. Am. J. Physiol.

[25] Pourahmad J, Amirmostofian M, Kobarfard F, Shahraki J (2009). Biological reactive intermediates that mediate dacarbazine cytotoxicity. Cancer. Chemother. Pharmacol.

[26] Novak RF, Kharasch ED, Wendel NK (1988). Nitrofurantoin stimulated proteolysis in human erythrocytes: a novel index of toxic insult by nitroaromatics. J. Pharmacol. Exp. Ther.

[27] Sakahira H, Enari M, Nagata S (1998). Cleavage of CAD inhibitor in CAD activation and DNA degradation during apoptosis. Nature.

[28] Lim MS, Lim PL, Gupta R, Boelsterli UA (2006). Critical role of free cytosolic calcium, but not uncoupling, in mitochondrial permeability transition and cell death induced by diclofenac oxidative metabolites in immortalized human hepatocytes. Toxicol. Appl. Pharmacol.

[29] Boelsterli UA (2003). Diclofenac-induced liver injury: a paradigm of idiosyncratic drug toxicity. Toxicol. Appl. Pharmacol.

[30] Tuschl G, Lauer B, Mueller SO (2008). Primary hepatocytes as a model to analyze species-specific toxicity and drug metabolism. Expert. Opin. Drug. Metab. Toxicol.

[31] Tugwood JD, Montague CT (2002). Biology and toxicology of PPARgamma ligands. Hum. Exp Toxicol.

[32] He K, Talaat RE, Pool WF, Reily MD, Reed JE, Bridges AJ, Woolf TF (2004). Metabolic activation of troglitazone: identification of a reactive metabolite and mechanisms involved. Drug. Metab. Dispos.

[33] Pompella A, Visvikis A, Paolicchi A, De Tata V, Casini AF (2003). The changing faces of glutathione, a cellular protagonist. Biochem. Pharmacol.

[34] Rezaei M, Rasekh HR, Ahmadiani A, Pourahmad J (2008). Involvement of subcellular organelles in inflammatory pain-induced oxidative stress and apoptosis in the rat hepatocytes. Arch. Iran. Med.

[35] Banos G, Reyes PA (1989). A comparative study of the effect of ten non-steroidal anti inflammatory drugs (NSAIDs) upon some mitochondrial and platelet functions. Int. J. Biochem.

[36] Uyemura SA, Santos AC, Mingatto FE, Jordani MC, Curti C (1997). Diclofenac sodium and mefenamic acid: Potent inducers of the membrane permeability transition in renal cortex mitochondria. Arch. Biochem. Biophys.

[37] Masubuchi Y, Nakayama S, Horie T (2002). Role of Mitochondrial Permeability Transition in Diclofenac- Induced Hepatocyte Injury in Rats. Hepatology.

[38] Boelsterli U, Priscilla L (2007). Mitochondrial abnormalities: A link to idiosyncratic drug hepatotoxicity. Toxicol. Appl. Pharmacol.

[39] Kowaltowski AJ, Castilho RF, Vercesi AE (1996). Opening of the mitochondrial permeability transition pore by uncoupling or inorganic phosphate in the presence of Ca2+ is independent of mitochondrial generated reactive oxygen species. FEBS. Lett.

[40] Pourahmad J, O’Brien PJ (2000). A comparison of hepatocyte cytotoxic mechanisms for Cu2+and Cd2+. Toxicology.

[41] Boelsterli UA, Boelsterli UA (2007). Xenobiotict-induced oxidative stress: Cell injury, signaling, and gene regulation. Mechanistic Toxicology: The Molecular Basis of How Chemicals Disrupt Biological Targets, 2nd ed.

[42] Bove Boveris A, Fraga CG, Varsavsky AI, Koch OR (1983). Increased chemiluminescence and superoxide production in the liver of chronically ethanol-treated rats. Arch. Biochem. Biophys.

[43] Ekström G, Ingelman-Sundberg M (1989). Rat liver microsomal NADPH-supported oxidase activity and lipid peroxidation dependent on ethanol-inducible cytochrome P-450 (P-450IIE1). Biochem. Pharmacol.

[44] Rashba-Step J, Turro NJ, Cederbaum AI (1993). Increased NADPH and NADH-dependent production of superoxide and hydroxyl radical by microsomes after chronic ethanol treatment. Arch. Biochem. Biophys.

